# Overexpression of *Arabidopsis thaliana* brassinosteroid-related acyltransferase 1 gene induces brassinosteroid-deficient phenotypes in creeping bentgrass

**DOI:** 10.1371/journal.pone.0187378

**Published:** 2017-10-30

**Authors:** Yun-Jeong Han, Young Soon Kim, Ok-Jin Hwang, Jeehee Roh, Keya Ganguly, Seong-Ki Kim, Ildoo Hwang, Jeong-Il Kim

**Affiliations:** 1 Department of Biotechnology and Kumho Life Science Laboratory, Chonnam National University, Gwangju, Republic of Korea; 2 Department of Life Science, Chung-Ang University, Seoul, Republic of Korea; 3 Department of Life Sciences and Biotechnology Research Center, Pohang University of Science and Technology, Pohang, Republic of Korea; RIKEN Center for Sustainable Resource Science, JAPAN

## Abstract

Brassinosteroids (BRs) are naturally occurring steroidal hormones that play diverse roles in various processes during plant growth and development. Thus, genetic manipulation of endogenous BR levels might offer a way of improving the agronomic traits of crops, including plant architecture and stress tolerance. In this study, we produced transgenic creeping bentgrass (*Agrostis stolonifera* L.) overexpressing a BR-inactivating enzyme, *Arabidopsis thaliana* BR-related acyltransferase 1 (AtBAT1), which is known to catalyze the conversion of BR intermediates to inactive acylated conjugates. After putative transgenic plants were selected using herbicide resistance assay, genomic integration of the *AtBAT1* gene was confirmed by genomic PCR and Southern blot analysis, and transgene expression was validated by northern blot analysis. The transgenic creeping bentgrass plants exhibited BR-deficient phenotypes, including reduced plant height with shortened internodes (i.e., semi-dwarf), reduced leaf growth rates with short, wide, and thick architecture, high chlorophyll contents, decreased numbers of vascular bundles, and large lamina joint bending angles (i.e., erect leaves). Subsequent analyses showed that the transgenic plants had significantly reduced amounts of endogenous BR intermediates, including typhasterol, 6-deoxocastasterone, and castasterone. Moreover, the AtBAT1 transgenic plants displayed drought tolerance as well as delayed senescence. Therefore, the results of the present study demonstrate that overexpression of an Arabidopsis BR-inactivating enzyme can reduce the endogenous levels of BRs in creeping bentgrass resulting in BR-deficient phenotypes, indicating that the *AtBAT1* gene from a dicot plant is also functional in the monocot crop.

## Introduction

Steroidal plant hormones, brassinosteroids (BRs), play important roles for various processes during plant growth and development, which include seed germination, cell division and elongation, vascular differentiation, plant architecture, reproduction, senescence, and responses to stresses [1–5]. Accordingly, the manipulation of endogenous levels of BRs has been widely used to improve the cultivation of crops, with effects on variables such as plant architecture, seed yield, and tolerance to stresses [6–10]. For example, BRs affect many agricultural traits that influence grain yield in rice, including plant height, leaf angle, grain size, and tiller number [11–13]. In addition, previous studies have shown that BR-deficient mutants display characteristic phenotypes such as dwarf, altered leaf morphology, abnormal vascular development, and delayed senescence [14–17]. On the other hand, excessive application of BRs down-regulates BR-biosynthesis genes and up-regulates BR-inactivation genes, hampering normal plant development. Therefore, the genetic manipulation of BR levels is suggested to offer a possibility for improving the agricultural traits of crops.

BRs are unlikely to undergo long-distance transport, because it has been shown that the sites of synthesis and action are same [11, 18]. Thus, mechanisms to modulate the levels of endogenous BRs in cells or tissues are essential to ensure appropriate growth and development of plants. For example, there exists negative feedback regulation in which the expression of BR biosynthesis and signaling genes is inhibited by BR treatment [16, 19, 20]. In addition, several modification processes are involved in BR inactivation, such as hydroxylation, sulfonation, glucosylation, and acylation. For example, cytochrome P450 monooxygenases, BAS1 (*phyB* activation-tagged suppressor 1) and SHK1 (Shrink1-D)/SOB7 (Suppressor of *phyB-4* 7)/CHI2 (CHIBI2) are involved in BR hydroxylation [21–25]. Steroid sulfotransferases, BNST3 and BNST4 from *Brassica napus* and AtST4a and AtST1 from *Arabidopsis thaliana*, have been reported to mediate BR sulfonation [26, 27]. UDP-glycosyltransferases, such as UGT73C5 and UGT73C6, have also been shown to catalyze the conjugation of BRs to glucose [28, 29]. More recently, it has been suggested that an acyltransferase, BAT1 (BR-related acyltransferase 1)/DRL1 (dwarf and round leaf 1)/PIZ (PIZZA) from *A*. *thaliana*, is involved in the conversion of active BR intermediates into inactive acylated conjugates [30–32]. Those studies suggest that the biosynthesis and inactivation of BRs are critical components for maintaining the endogenous levels in plants, which is important for the regulation of plant growth and development.

Creeping bentgrass (*Agrostis stolonifera* L.) is an economically important turfgrass species used on golf courses extensively [33]. However, because of its vigorous growth and intolerance to drought stress, frequent mowing and daily irrigation are often required to cultivate creeping bentgrass plants. Thus, it is desirable to reduce the frequency of mowing and to increase drought tolerance in creeping bentgrass. In this regard, genetic transformation utilizing a target gene can be an effective way to produce turfgrass varieties with a reduced growth rate to lower the mowing frequency and/or tolerance to drought stress. The target genes that may be used for this transformation include those related to BRs, because they are suggested as key components for improving the quality of agricultural products [6, 34, 35]. In this study, we aimed to produce and analyze transgenic turfgrass plants with reduced levels of endogenous BRs using a BR-inactivating gene, *A*. *thaliana BAT1* (*AtBAT1*). For this, we generated transgenic creeping bentgrass plants overexpressing the *AtBAT1* gene under the control of cauliflower mosaic virus (CaMV) 35S promoter, and demonstrated that the transgenic plants displayed BR-deficient phenotypes, such as semi-dwarf growth, with reduced levels of endogenous BR intermediates. More interestingly, further phenotypic analyses confirmed drought tolerance of the transgenic plants, compared with non-transformed wild-type creeping bentgrass plant. Therefore, these results suggest that the *AtBAT1* gene from a dicot plant is functional in the monocot crop, and provide a method to develop turfgrass varieties with shortened height and improved tolerance to drought stress.

## Materials and methods

### Gene construct

Total RNA was extracted from *A*. *thaliana* (L*er* ecotype) seedlings using TRIzol^®^ reagent (Invitrogen, CA) and cDNA was synthesized from 2 μg of total RNA using the MMLV-reverse transcriptase (Promega, WI), according to the manufacturer’s instructions. The *AtBAT1* gene (At4g31910) was then PCR-amplified from the cDNA using primers, 5′- TGCTCTAGAATGCCCATGTTAATGGCGACACGT-3′ (forward, with an *Xba*I site) and 5′- TCCCCCGGGTTAGCAATCAAGGAAATGATTTGA-3′ (reverse, with a *Sma*I site), and cloned into a binary vector pCAMBIA3300, with *Xba*I and *Sma*I. The CaMV 35S promoter and the *NOS* transcriptional terminator were used to drive the expression of *AtBAT1*. The *BAR* gene for herbicide resistance in the binary vector was used as a selectable marker to obtain putative transgenic creeping bentgrass plants. The integrity of the construct was confirmed by DNA sequencing, and then used to transform *Agrobacterium tumefaciens* strain EHA105 using the freeze-thaw method [36], followed by creeping bentgrass transformation.

### Generation of putative transgenic creeping bentgrass plants

Seeds for the ‘‘Crenshaw” cultivar of creeping bentgrass (*A*. *stolonifera* L.) were purchased from KVBio Inc., Korea, and stored at 4°C before use. Tissue culture and genetic transformation of creeping bentgrass were performed as previously described [37, 38]. After transformation, plantlets with well-developed roots were transferred to soil, grown under greenhouse conditions for 2 weeks, and then sprayed with 0.4% (v/v) BASTA^®^ (containing 18% glufosinate ammonium) to select putative transgenic creeping bentgrass plants. Herbicide resistance was determined 10 days later, and herbicide-resistant plants were further analyzed.

### Molecular analysis of transgenic creeping bentgrass plants

Genomic PCR, Southern blot, and northern blot analyses were performed as previously described [39–41]. For genomic PCR analysis, total genomic DNA was isolated from the leaves of mature plants and the coding regions for *AtBAT1* and *BAR* transgenes were PCR-amplified. The primers used for *BAR* were 5′-CTACCATGAGCCCAGAACGACG-3′ (forward) and 5′-CTGCCAGAAACCCACGTCATGCCAGTTC-3′ (reverse). In addition, the actin gene (*ACT*) of creeping bentgrass was also PCR-amplified using the same template and the primers 5′-AACTGGGACGACATGGAGAAGATA-3′ (forward) and 5′-CGTCAGGGAGCTCGTAGTTCTTC-3′ (reverse), which is included as a loading control for genomic DNA. For Southern blots, 30 μg of each genomic DNA sample was digested with either *Xba*I, *EcoR*I or *Hin*dIII, and hybridizations were performed with the *BAR* gene probe labeled with [α^32^P] dCTP using the Radiprime^™^ II Random Prime Labeling System (Amersham Biosciences, UK). For northern blots, 15 μg of total RNA was extracted from leaves using the TRIzol^®^ reagent, and hybridizations were carried out with a [α^32^P] dCTP-labeled *BAR or AtBAT1* probe. Hybridization signals were detected via the exposure on X-ray films (Amersham Biosciences, UK). For these analyses, non-transformed creeping bentgrass plant (NT) and a transgenic plant with an empty vector, pCAMBIA3301 (HR, i.e., the herbicide-resistant creeping bentgrass reported in [40]) were included as control plants.

### Phenotypic analysis of transgenic creeping bentgrass plants

Creeping bentgrass plants in soil were grown and vegetative propagated routinely in a culture room (22–24°C with a 16 h photoperiod). For the phenotypic analysis, the plants were grown to similar stages and trimmed to the same heights, and then the trimmed plants were further grown for 4 weeks in the culture room. Plant height was measured as the length from the ground to the top of each plant using a ruler. Internode and leaf lengths were measured from the second leaf of the first stoloniferous plant using a ruler, and leaf width was measured on the widest part of the second leaf using a vernier caliper. In addition, leaf thickness was measured from the cross-sections of the second leaves using ocular micrometer under a microscope.

To investigate the growth rate of creeping bentgrass plants, leaf growth rates were evaluated by measuring leaf lengths every 5 days during 5 weeks of growth. Five leaves of each plant were used for the measurements, and the leaf growth rate (cm/5 days) was calculated from the following: [(the leaf length of current measurement)–(the leaf length of the previous measurement)] at 5-day intervals. All measurements were repeated at least three times and the results were consistent.

### Microscopic analysis of cross-sections

Cross-sections and microscopic analyses were performed as previously described [42]. The fully expanded second leaves or internodes were collected and fixed in formalin-acetic acid-alcohol, followed by dehydration with graded ethanol, and infiltration in catalyzed resin (1.25 g of benzoyl peroxide per 100 mL of immunobead monomer A). Samples were then embedded, polymerized at room temperature, and placed in a desiccator under a vacuum until they were ready to block. Photographs were then taken using a microscope, and the number of veins in leaves was counted. In addition, cell length was measured from longitudinal sections of xylem cells in the second internodes using ocular micrometer under a microscope.

### Measurements of chlorophyll contents

After growing for 4 weeks in the culture room, the second leaves of the first stoloniferous plants were harvested. Then, 300 mg of fresh leaves was homogenized and extracted with buffered 80% aqueous acetone (pH 7.8). The chlorophyll (chl) content was estimated as described previously [43]. Chlorophyll *a* and *b* contents (mg/g) were calculated from the equations, [(12.70 × λ_663_ − 2.69 × λ_645_) × V/1000 × FW] and [(22.9 × λ_645_ − 4.86 × λ_663_) × V/1000 × FW], respectively, where V is the volume of the extract (mL) and FW is the fresh weight of leaves (g). Total chlorophyll (mg/g) was also calculated from the equation, [(8.02 × λ_663_ + 20.20 × λ_645_) × V/1000 × FW].

For immunoblots that were used to detect light-harvesting chlorophyll-binding (Lhcb) proteins, leaves from each plant were ground in extraction buffer (70 mM Tris-HCl, pH 8.3, 7 mM EDTA, 35% ethylene glycol, 98 mM ammonium sulfate, 14 mM sodium metabisulfite, 0.07% polyethyleneimine, and 2.8 mM PMSF). Then, 30 μg of each protein extract was loaded onto 10% SDS-PAGE gels and transferred to a polyvinylidene difluoride membrane (Hybond-P; Amersham Biosciences, UK). The membrane was incubated with specific antibodies against Lhcb proteins (Santa Cruz Biotechnology, CA) and developed using an ECL^™^ western blotting analysis system (Amersham Biosciences, UK).

### Quantitative analysis of endogenous BRs in creeping bentgrass

Lyophilized leaf samples of 5-week-old plants were extracted three times with 500 mL of 90% methanol. [26, 28-^2^H_6_]-6-deoxocastasterone (6-deoxoCS), [26, 28-^2^H_6_]-typhasterol (TY), [26, 28-^2^H_6_]-CS and [26, 28-^2^H_6_]-brassinolide (BL) were added as internal standards to allow the quantitative analysis of the extracts. After evaporation, the extracts were partitioned three times between distilled water and chloroform (1:1 ratio). The chloroform fractions were further partitioned between 80% methanol and n-hexane (1:1), and the 80% methanol fractions were repartitioned between ethyl acetate and sodium phosphate buffer of pH 7.8 (1:1). Then, the obtained ethyl acetate fractions were subjected to silica gel (25 g) chromatography. The column was eluted with 200 mL of chloroform containing 1, 3, 5, 7, 10, 50, and 100% methanol. The 5 and 7% methanol fractions were combined, concentrated, and purified using a SepPak C18 cartridge column (10 g, Waters Co.) by eluting with 50 mL of 50, 60, 90, and 100% methanol. The 90% methanol fractions were further purified by a reverse phased HPLC (SenshuPak C18, 10 × 150 mm), eluting at a flow rate of 2.5 mL min^-1^ using acetonitrile-water gradients: 45% acetonitrile (0 to 20 min), 45 to 100% acetonitrile (20 to 40 min), and 100% acetonitrile (40 to 70 min). Under the same HPLC conditions, BL, CS, and TY/6-deoxoCS were detected in fractions 15 to 17, 22 to 23, and 37 to 39, respectively. The correspondent HPLC fractions relating to authentic BRs were further analyzed by a capillary GC-MS/SIM (Gas Chromatography-Mass Spectroscopy/Selective Ion Monitoring).

GC-MS/SIM analyses were performed using a Hewlett-Packard 5973 mass spectrometer (electron impact ionization, 70 eV) connected to 6890 gas chromatography fitted with a fused silica capillary column (HP-5, 0.25 mm × 15 m, 0.25-μm film thickness). The oven temperature was maintained at 175°C for 2 min, elevated to 275°C at a rate of 40°C min^-1^, and then maintained at 275°C for 15 min. Helium was used as the carrier gas at a flow rate of 1 mL min^-1^, and samples were introduced using an on-column injection mode. 6-deoxoCS, CS, and BL were analyzed as bismethaneboronates, and TY was analyzed as monomethaneboronate-trimethylsilyl ether. The amounts of BRs in plants were calculated from the relative intensities of molecular ions for endogenous BRs against those for [26, 28-^2^H_6_]-BRs added as internal standards.

### Lamina joint bending assay

The angle of leaf bending was estimated by lamina inclination assays as described previously [44]. Creeping bentgrass plants were grown to a similar stage, trimmed to the same height, and grown for further 4 weeks in the culture room. Then, the angles between the second leaf lamina and the sheath were measured from the captured images using the ImageJ program.

### Leaf senescence and drought tolerance assay

For leaf senescence assay, the detached leaves were incubated for 2 weeks in the dark at 25°C, and losses in chlorophyll content was measured. The percentage of chlorophyll loss was calculated as follows: [(total chlorophyll before senescence treatment) − (total chlorophyll remained after treatment)]/(total chlorophyll before treatment) × 100.

For drought tolerance assay, creeping bentgrass plants were grown to a similar stage in 30 × 20 cm plastic trays containing the same amount of mixture (peat moss: vermiculite: perlite = 3:2:1), and were subjected to drought stress. All pots were fully irrigated before the start of the drought treatment, and then drought stress was induced by terminating irrigation. Sixteen days after irrigation was terminated, the plants were re-irrigated, and drought tolerance was examined after 10 days by calculating the survival rate, which was determined by counting the number of surviving plants out of all plants after re-irrigation. The drought tolerance assays were repeated at least three times with consistent results. In addition, leaf relative water content (RWC) was estimated as the percentage from (fresh weight—dry weight)/(turgid weight—dry weight) × 100, as described previously [45].

### Measurement of stomatal closure

To investigate responses to ABA in creeping bentgrass, stomatal closing was measured in the absence or presence of ABA [stock solution in dimethyl sulfoxide (DMSO)], as previously described [39]. For this, epidermal strips were peeled off from three-week-old leaves using tissue forceps and incubated in a buffer (10 mM Mes/KOH, 50 mM KCl, pH 6.1) at 22°C under white light condition. 2 h after the incubation, the stomata were further incubated for 2 h in the absence (0.1% DMSO only) or presence of 50 μM ABA, and then photographed using a Olympus BX51 microscope equipped with an UPIanF1 40X/0.75 objective lens and a WH10X/22 ocular lens. The stomata were observed as three types (completely open, partially open, and completely closed), and the number of completely closed stomata was counted from 150 guard cells in each plant to calculate the percentages of stomatal closing.

### Statistical analysis

IBM SPSS statistics 20 software was used to perform ANOVA to analyze the results of the physiological parameters. A significant difference from the control value was determined at *P* < 0.05 (marked with *) or *P* < 0.001 level (marked with **). All data represented the mean ± standard deviation (SD) of at least three independent experiments.

## Results

### Production of transgenic creeping bentgrass plants overexpressing *AtBAT1*

**Fig 1:**
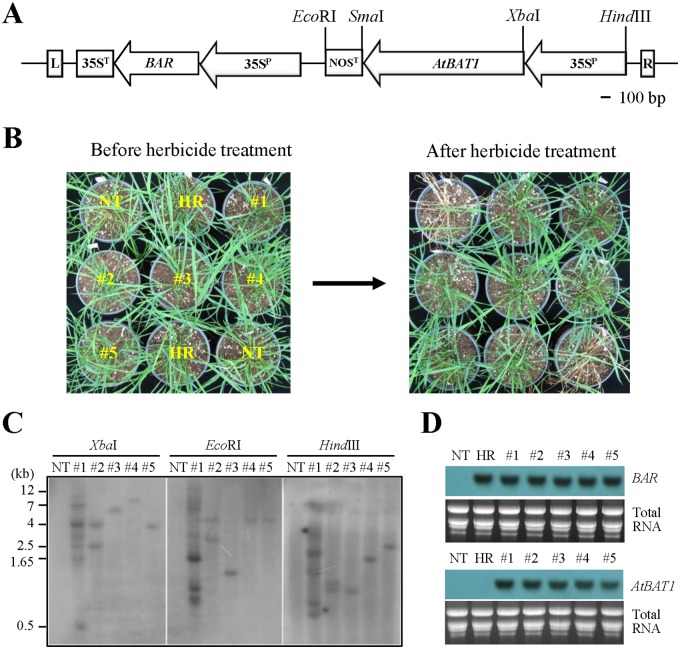
Production of transgenic creeping bentgrass plants overexpressing *AtBAT1*. (A) T-DNA region of the binary vector pCAMBIA3300 harboring the *AtBAT1* expression cassette. R, right border; L, left border; 35S^p^, CaMV 35S promoter; 35S^T^, 35S transcriptional terminator; NOS^T^, *NOS* transcriptional terminator; *BAR*, phosphinothricin acetyltransferase gene; *AtBAT1*, *Arabidopsis thaliana* BR-related acyltransferase 1 gene. (B) Herbicide resistance assay. NT, non-transformed creeping bentgrass plant; HR, transgenic creeping bentgrass plant with an empty vector (i.e., a positive control plant with herbicide resistance). Numbers represent putative transgenic creeping bentgrass lines carrying *AtBAT1*. Herbicide resistance was determined 10 days after 0.4% BASTA^®^ treatment. (C) Southern blot analysis. Genomic DNA (30 μg) from each plant was digested with *Xba*I, *Eco*RI, or *Hind*III, and then hybridized with the *BAR* probe. (D) Northern blot analysis. Total RNA (15 μg) from each plant was used for hybridizations with the *BAR* or *AtBAT1* probe. Total RNA is shown as loading controls.

To generate transgenic plants, the *AtBAT1* gene was introduced into creeping bentgrass (cv. Crenshaw) through *Agrobacterium*-mediated transformation. For this, mature seeds-derived embryogenic calli and the binary vector pCAMBIA3300 harboring the *AtBAT1* gene expression cassette under the control of 35S promoter and *NOS* terminator (Fig 1A) were used for the transformation using the method that we established for creeping bentgrass [37–41, 46, 47]. Putative transgenic creeping bentgrass plants were obtained by spraying with 0.4% BASTA^®^ herbicide. Non-transformed control plant (hereafter, NT plant; a negative control) died within 10 days after the herbicide treatment, whereas a transgenic bentgrass plant carrying an empty vector (hereafter, HR plant; a positive control) and all putative transformants exhibited resistance (Fig 1B). These results indicate that the *BAR* transgene is expressed in the transgenic plants. Thus, further molecular analyses were conducted with the obtained transgenic lines.

To confirm the insertion of the *BAR* and *AtBAT1* transgenes in the transgenic plants, genomic PCR analysis was performed with10 transgenic plants [two plants from five independent transgenic lines (#1~5)] (S1 Fig). Integration of transgenes was observed in all putative transgenic plants that exhibited herbicide resistance, whereas no amplified band was detected in the NT plant. In the HR plant, a band was amplified for *BAR*, but not for *AtBAT1*. The transgene integration was further verified by Southern blots using a *BAR*-specific probe (Fig 1C). Southern blot analysis of five transgenic lines revealed that three lines (#3, #4, and #5) had single transgene integration but generated different patterns of bands in the blots, indicating that they are independent transgenic lines carrying one integrated gene. On the other hand, lines #2 and #1 showed two and multiple bands in the blots, respectively, suggesting the presence of multiple integrated genes. No hybridization signal was observed in the NT plant. The expression of both transgenes was then analyzed by northern blots using the *BAR* and *AtBAT1* probes (Fig 1D). The results showed that all transgenic plants expressed the integrated transgenes, while the NT plant did not show any hybridization signal. In the case of the HR plant, the expression of *BAR* was detected, but that of *AtBAT1* was not. Collectively, we obtained three independent transgenic lines with single transgene integrations, which expressed at similar levels of *AtBAT1*. Therefore, those lines (#3, #4, and #5) were selected for further phenotypic analysis.

### Transgenic plants carrying *AtBAT1* display dwarf phenotypes with a reduced growth rate

**Fig 2:**
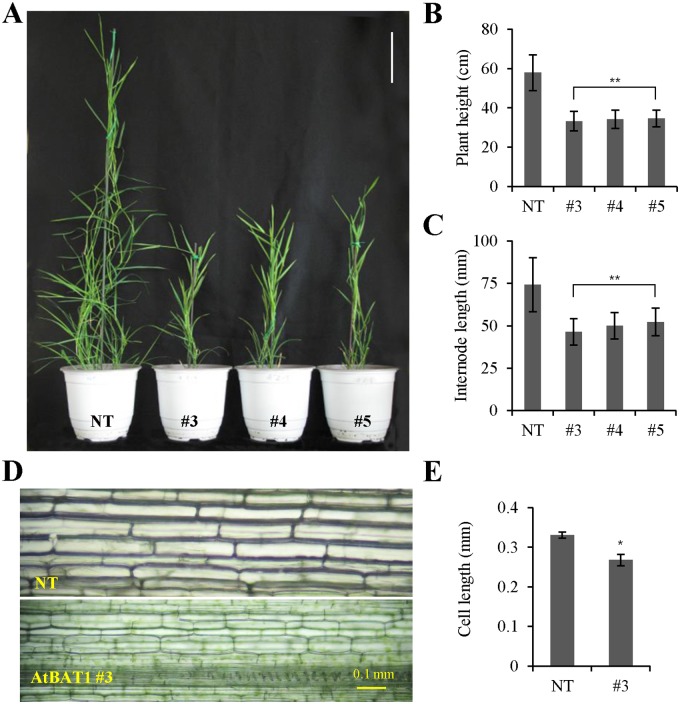
Semi-dwarf phenotypes of transgenic creeping bentgrass plants with *AtBAT1*. (A) Fully-grown creeping bentgrass plants. NT, non-transformed control plant; #3, #4, and #5, transgenic plants overexpressing *AtBAT1*. The plants were grown to similar stages, trimmed to the same heights, and then the trimmed plants were grown for 4 weeks. Bar = 10 cm. (B,C) Measurements of plant heights and internode lengths. Plant height was measured as the length from the ground to the top of each plant, and internode length was measured from the second leaf of the first stoloniferous plant. Data represent means ± SD of three independent measurements (*n* = 30 for each measurement). (D) Longitudinal sections displaying plant cells in the second internodes. Bar = 0.1 mm. (E) Measurement of cell lengths in (D). Data represent means ± SD (*n* = 10). Statistically significant changes compared with NT are indicated by * at *P* < 0.05 or ** at *P* < 0.001.

Transgenic creeping bentgrass plants overexpressing *AtBAT1* (hereafter, AtBAT1 plants) were grown in a greenhouse, and their phenotypes were analyzed by comparing with the NT plant. First, a semi-dwarf phenotype was observed in the AtBAT1 plants (Fig 2A). Under the growth conditions, the average height of 4-week-grown NT plant was about 57.8 cm, whereas that of the AtBAT1 plants was approximately 34.0 cm (Fig 2B). The average internode length of the AtBAT1 plants was 49.5 mm compared with 74.2 mm in the NT plant (Fig 2C). When cells from longitudinal sections of the second internodes were observed, the cell lengths were found to be significantly decreased in the transgenic line #3, compared to those in the NT plant (Fig 2D and 2E). This might explain the reduced internode lengths, which may lead to shortened heights. Since three independent transgenic plants showed very similar semi-dwarf phenotypes, these results suggest that the observed phenotype is due to the expression of *AtBAT1* in creeping bentgrass.

**Fig 3:**
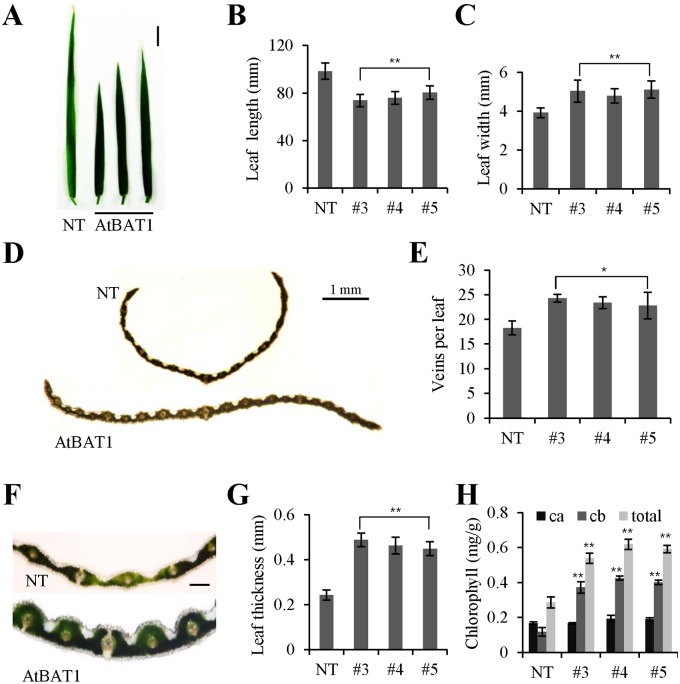
Leaf phenotypes of transgenic creeping bentgrass plants with *AtBAT1*. (A) Representative leaves from non-transformed (NT) and transgenic plants (AtBAT1). The second leaves from the main branch of 4-week-grown plants are shown. Bar = 1 cm. (B, C) Measurements of leaf lengths and widths. Leaf length was measured from the second leaf of the first stoloniferous plant and leaf width was measured from the widest part from the second leaf. Data represent means ± SD of three independent measurements (*n* = 20 for each measurement). (D) Cross-sections of the second leaves in full length. Bar = 1.0 mm. (E) Measurement of vein numbers per leaf. Data represent means ± SD (*n* = 10). (F) Cross-sections of the second leaves. Bar = 0.2 mm. (G) Measurement of leaf thicknesses. (H) Measurement of chlorophyll contents. ca, chlorophyll *a*; cb, chlorophyll *b*; total, total chlorophylls. Data represent means ± SD of three independent measurements. Statistically significant changes compared with NT are indicated by * at *P* < 0.05 or ** at *P* < 0.001.

Next, we investigated leaf phenotypes, and found that the leaves of the AtBAT1 plants were shorter, wider, thicker, and greener than those of the NT plant (Fig 3A). The average leaf length of 4-week-grown NT plant was about 9.8 cm, whereas that of the AtBAT1 plants was approximately 7 to 8 cm (Fig 3B). In contrast, the leaf width of the AtBAT1 plants was increased when compared with that of NT plant (4.7~5.1 *vs*. 3.9; Fig 3C). When we observed the cross-sections of leaves, we found that the number of veins was increased in the transgenic plants compared with the NT plant (Fig 3D and 3E). This might explain why the AtBAT1 plants had wider leaves. In addition, the leaves of the AtBAT1 plants were approximately two-fold thicker than those of the NT plant (Fig 3F and 3G). Moreover, chlorophyll contents in the leaves of the AtBAT1 plants were significantly higher than those in the leaves of the NT plant (Fig 3H). It is notable that the levels of chlorophyll *b*, but not chlorophyll *a*, were increased in the transgenic plants, which contributed to the increase in total chlorophyll contents. We also found that the chlorophyll contents did not differ between young leaves of the NT and transgenic plants, whereas the chlorophyll contents in mature leaves were quite different, suggesting that more chlorophyll accumulates in transgenic plants during growth than in the control plant (S2A Fig). To confirm the high accumulation of chlorophylls in the AtBAT1 plants, we conducted western blot analysis to determine the expression of the light-harvesting chlorophyll-binding (Lhcb) proteins using leaf samples from 4-week-grown plants. The results showed that all four Lhcb proteins tested in this study accumulated to higher levels in the AtBAT1 plants than in the NT plant (S2B Fig).

**Fig 4:**
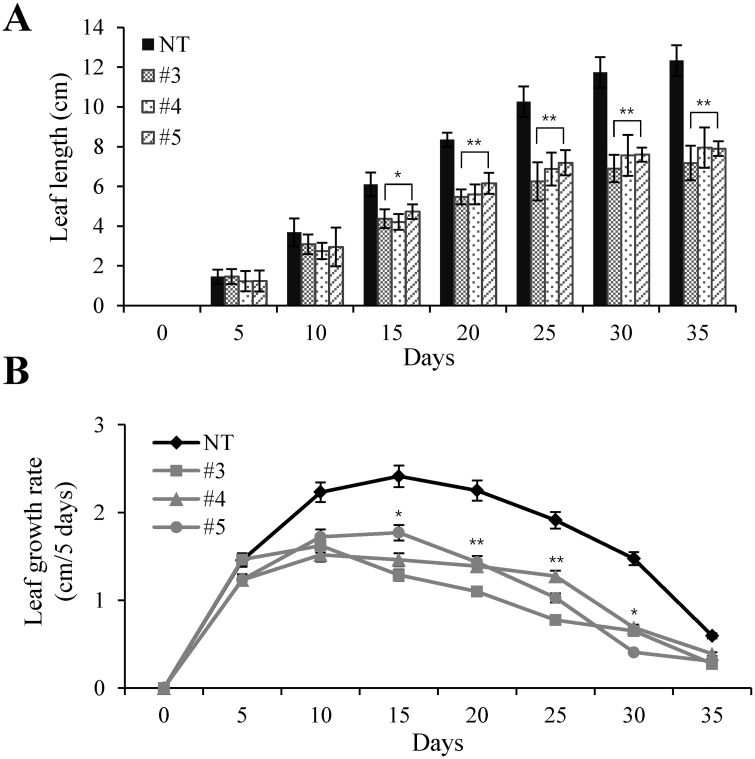
Leaf growth rates of the AtBAT1 plants. (A) Growth-time dependent measurements of leaf growth. The leaf lengths were measured for 5 weeks at 5-day intervals. (B) Measurements of leaf growth rates. Leaf growth rates (cm/5 days) were calculated by subtracting the leaf length at previous measurement from the leaf length at current measurement at 5-day intervals. Data represent means ± SD of three independent measurements (*n* = 8 for each measurement). Statistically significant changes compared with NT are indicated by * at *P* < 0.05 or ** at *P* < 0.001.

To further examine whether the dwarf phenotype is due to growth retardation, we investigated the leaf growth rates by measuring leaf lengths in a time-dependent manner. Under our growth conditions, the leaf growth rate of the NT plant increased up to 15 days and then decreased (Fig 4). Similarly, the maximum leaf growth rate of the AtBAT1 plants was observed about 15 days after growth. However, the magnitude of the growth rates was significantly reduced in the AtBAT1 plants compared with the NT plant (Fig 4B). Therefore, these results suggest that the transgenic plants overexpressing *AtBAT1* display a semi-dwarf phenotype due to the reduced growth rate. The results obtained with the AtBAT1 plants correlated well with the phenotypes of BR-deficient (loss-of-function in BR biosynthesis) or BR-insensitive (loss-of-function in BR perception or signaling) mutants, which usually display dwarf phenotypes with compact statures, reduced cell elongation, and smaller leaves with dark green color compared than wild-type control plant [48, 49].

### Overexpression of *AtBAT1* decreases endogenous BR intermediates in creeping bentgrass, displaying BR-deficient phenotypes

**Fig 5:**
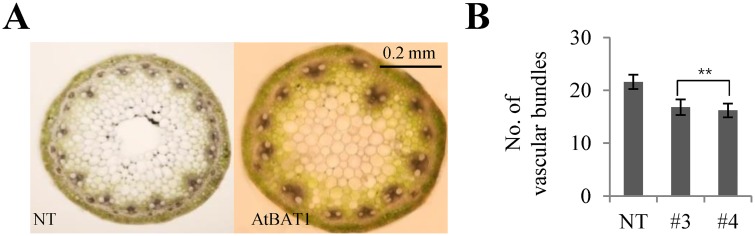
Comparison of vascular bundles in internodes between control plant (NT) and the AtBAT1 plants. (A) Cross-sections of internodes from the second leaf of the first stoloniferous plant. (B) Measurements of the number of vascular bundles. Data represent means ± SD of three independent measurements (*n* = 20 for each measurement). Statistically significant changes compared with NT are indicated by ** at *P* < 0.001.

The *AtBAT1* gene was originally isolated from a mutant with altered vascular bundle development using the FOX (full-length cDNA overexpressor) hunting system [30]. The Arabidopsis mutant overexpressing *AtBAT1* showed a dwarf phenotype with fewer vascular bundles in its inflorescence stem compared to the wild-type stem. Thus, we further investigated vascular bundles from cross-sections of the internodes. The results showed that the number of vascular bundles in the AtBAT1 plants was reduced compared with those in the NT plant (Fig 5). These results suggest that AtBAT1 functions similarly in both Arabidopsis and creeping bentgrass.

In addition, BR profile analysis using Arabidopsis plants revealed that *AtBAT1* overexpression substantially reduced the endogenous levels of several BR biosynthetic intermediates, especially the BRs with α-configuration at C3-hydroxy group such as typhasterol (TY), 6-deoxoTY, castasterone (CS), and 6-deoxoCS [30–32]. Thus, we investigated endogenous levels of BRs in creeping bentgrass plants by GC-MS analysis using available internal standards, [26, 28-^2^H_6_]-labeled TY, CS, 6-deoxoCS, and brassinolide (BL). The results showed that endogenous BR intermediates were significantly reduced in the AtBAT1 plants, compared with the NT plant (Table 1). Endogenous levels of BR intermediates upstream of BL, such as TY, 6-deoxoCS, and CS, were decreased to approximately 45–53, 54–60, and 50–55% of wild-type levels, respectively. Active BL was not detectable in creeping bentgrass plants, as similar results were reported in other plants [30, 32, 50, 51]. Overall, these results suggest that overexpression of the *AtBAT1* gene alters the endogenous levels of BRs in creeping bentgrass, which might explain the semi-dwarf phenotypes observed in the AtBAT1 plants.

**Table 1:** Quantitative analysis of endogenous BR intermediates in creeping bentgrass.

BRs	NT	AtBAT1 #3	AtBAT1 #4
Typhasterol (TY)	7.36/6.86	3.22/3.31	3.46/3.91
6-deoxocastasterone (6-deoxoCS)	9.40/9.16	5.40/5.44	4.83/5.62
Castasterone (CS)	7.10/5.10	3.48/2.51	3.62/3.12
Brassinolide (BL)	n.d.	n.d.	n.d.

Endogenous BR levels were measured by GC-SIM using [26, 28-^2^H_6_]-labeled BRs as internal standards. The leaves of 5-week-old plants in soil were used in these analyses, and the contents (ng/g fresh weight) of BR intermediates are shown, which were obtained from two independent experimental measurements (shown as the first result/the second result). NT, Non-transformed creeping bentgrass; AtBAT1 #3 and #4, two transgenic creeping bentgrass plants overexpressing *AtBAT1*; n.d., not detected (i.e., below the detection limit).

**Fig 6:**
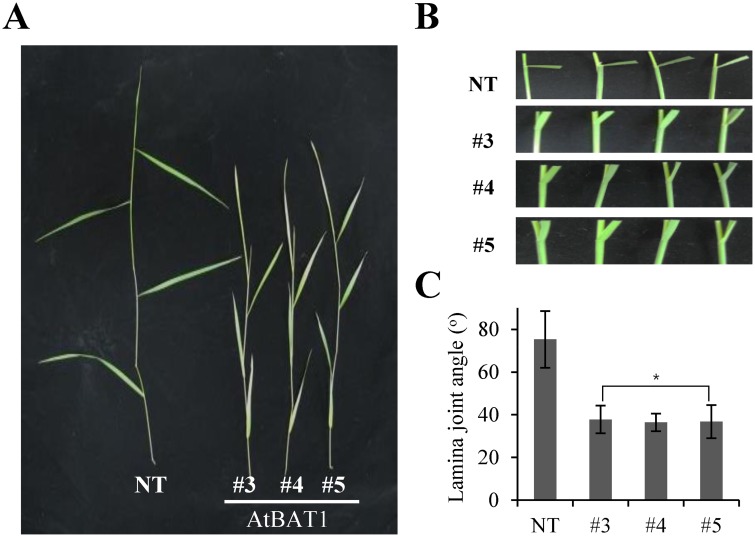
Leaf bending phenotypes of the AtBAT1 plants. (A) Representative leaves from 4-week-grown creeping bentgrass plants under greenhouse conditions. (B) Lamina inclination between leaf blade and vertical culm. (C) Measurement of lamina joint bending angles. The angle between the lamina and the sheath of the second leaf was measured from the captured images using ImageJ program. Data represent means ± SD (*n* = 10). Statistically significant changes compared with NT are indicated by * at *P* < 0.05.

Since BR profile analysis confirmed that BR levels were reduced in the AtBAT1 plants, we further investigated other BR-deficient phenotypes. One of the earliest reports of BR-mediated phenotypes in monocot plants was related to leaf bending or lamina inclination in rice [12, 52, 53]. Thus, we investigated the leaf bending of creeping bentgrass plants by measuring the inclination between the leaf blade and the vertical culm (i.e., lamina joint bending angles). The results showed that the lamina joint bending angles of the AtBAT1 plants were greater than those of the NT plant (Fig 6). The transgenic plants showed erect leaves with decreased angles (~37°) compared with the angle (~75°) of the NT plant (Fig 6C). Together, the present results clearly suggest that overexpression of *AtBAT1* induced BR-deficient phenotypes in creeping bentgrass with decreased endogenous BR intermediates.

### Transgenic plants with *AtBAT1* exhibit drought tolerance and delayed senescence

**Fig 7:**
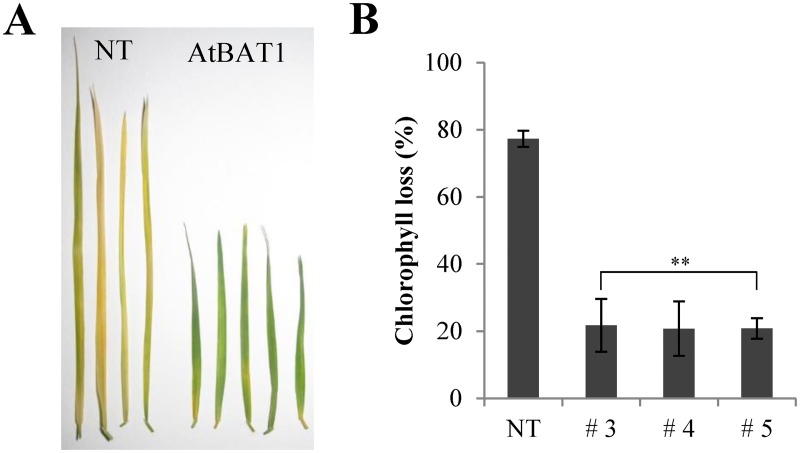
Comparison of leaf senescence between control plant (NT) and the AtBAT1 plants. (A) Leaves after senescence treatment. The detached leaves were incubated for 2 weeks in the dark at 25°C. (B) Loss of chlorophyll content after the senescence treatment. Data represent means ± SD of three independent measurements. Statistically significant changes compared with NT are indicated by ** at *P* < 0.001.

Leaf senescence might be one of important agronomic traits for turfgrass, and BRs have been known to regulate senescence [4, 54]. Thus, we further carried out leaf senescence assays with the AtBAT1 plants by measuring losses in chlorophyll content following senescence treatment (Fig 7). After incubation in the dark for 2 weeks, the leaves of the NT plant lost ~77% of their chlorophyll content, whereas those of the AtBAT1 plants lost ~21%. Thus, the AtBAT1 plants exhibit delayed leaf senescence, as observed in other BR-deficient phenotypes.

**Fig 8:**
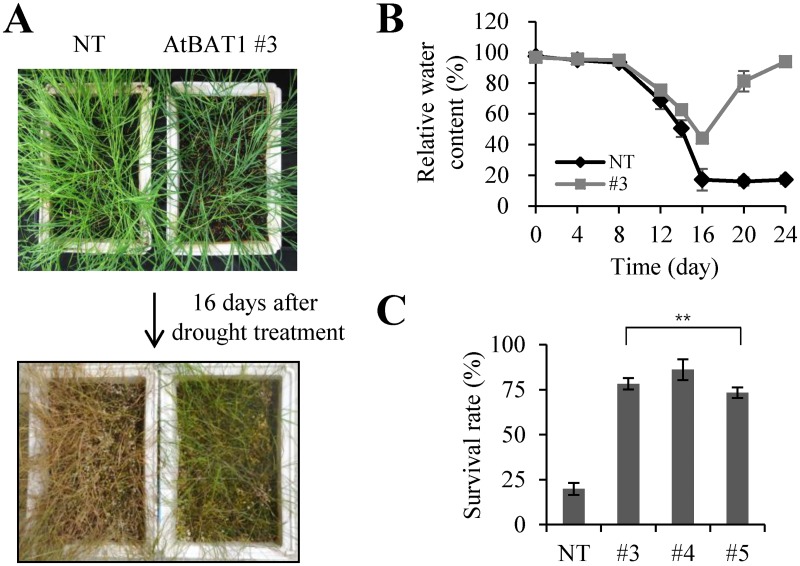
Drought tolerance of the AtBAT1 plants. (A) Drought tolerance assay. Water was withhold from 4-week-grown plants for 16 days, and images were taken before and after drought treatment. As the results of transgenic plants #4 and #5 were similar to those of #3, transgenic plant #3 is shown as a representative plant. (B) Measurements of relative water content (RWC). The leaves were harvested every 4 days after stopping irrigation, and the fresh, turgid, and dry weights were measured to calculate the RWC. Data represent mean ± SD of three independent measurements. (C) Average survival rates after drought treatment. Data represent means ± SD of three independent measurements (*n* = 60 for each measurement). Statistically significant changes compared with NT are indicated by ** at *P* < 0.001.

Drought tolerance is another desired agronomic trait for creeping bentgrass that is drought-intolerant as a cool season turfgrass, and previous studies have suggested that BRs play essential roles in responding to various stresses, including drought [2]. Thus, we compared drought tolerance of the AtBAT1 plants with the NT plant. After 16 days of drought treatment, most of the NT plants wilted, whereas the transgenic plants showed less wilting (Fig 8A). During the drought treatment, we also compared the relative water content (RWC) in leaves of the NT and transgenic plants (Fig 8B). The results showed that the estimated RWC was approximately 38–44% in the leaves of the AtBAT1 plants after 16 days of drought treatment, which was significantly higher than that in the leaves of the NT plant (~17%). These results indicate that the water content of the AtBAT1 plants was slowly reduced during the drought treatment, compared with the NT plants. Moreover, after re-watering, approximately 80% of the AtBAT1 plants survived, whereas only 20% of the NT plants survived (Fig 8C). These results suggest that the overexpression of *AtBAT1* might enhance drought tolerance in creeping bentgrass.

**Fig 9:**
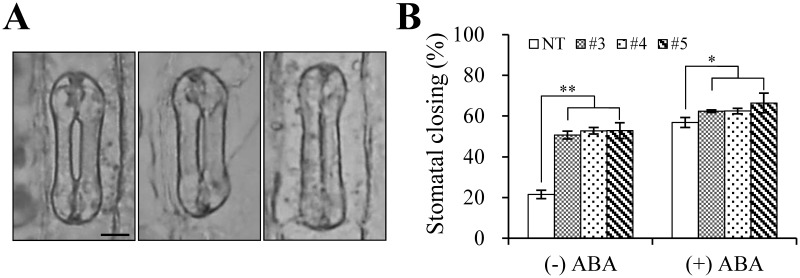
Stomatal closing analysis of the AtBAT1 plants. (A) Representative stomata observed in creeping bentgrass. The observed stomata could be classified into three types: completely open (left), partially open (middle), and completely closed (right). Bar = 5 μm. The apertures of the completely and partially open stomata are approximately 2.5 and 1.4 μm, respectively, whereas those of the completely closed stomata are not measurable. (B) The ratios of stomata closing in the absence (−) and presence (+) of ABA. The number of completely closed stomata was counted to calculate the percentages of stomatal closing. Data represent means ± SD of three independent measurements (*n* = 150 for each measurement).

The observed drought tolerance phenotype of the AtBAT1 plants was somewhat unexpected, because the treatment of plants with exogenous BRs has shown to increase tolerance to stresses [5, 9]. However, a recent study has shown that the reduced BR accumulation could improve drought tolerance, due to increased plant responsiveness to abscisic acid (ABA) [55]. Thus, we further investigated responses of the AtBAT1 plants to ABA by observing stomatal closing in the absence and presence of ABA (Fig 9). The results showed that the AtBAT1 plants exhibited a higher percentage of the closed stomata even in the light condition than the NT plant (~52% *vs*. ~22%), as well as in the condition with ABA treatment (~64% vs. ~57%) (Fig 9B). Compared to the NT plant, the higher ratios of stomatal closing in the AtBAT1 plants might explain the slower reduction of water content during the drought treatment, which might confer the plants tolerance to drought stress. Combined with the results in the recent report [55], the present results suggest that the reduced levels of endogenous BRs might make the plants to be hypersensitive to ABA, in turn, enabling them to be drought tolerant.

## Discussion

The *AtBAT1* gene (At4g31910), also known as *PIZ* and *DRL1*, was originally isolated in studies of mutants with decreased vascular bundles in their inflorescence stem [30], those displaying BR-deficient dwarf phenotypes with small round leaves of dark green color [31], and the mutant with short petioles and round leaves [32]. The Arabidopsis mutants were obtained by screening the FOX collection or activation-tagging lines, in which the mutation was caused by overexpression of the BR-related acyltransferase gene. On the other hand, T-DNA insertion mutants of the *AtBAT1* gene do not exhibit significant differences in morphology compared with the wild-type plant after flowering, although they showed longer and larger inflorescence stems before flowering and an increased number of vascular bundles [30]. Thus, overexpression of *AtBAT1* generated plants with significant phenotypes rather than deficiency, which might be due to redundancy of BR-inactivation mechanisms in addition to acylation, such as hydroxylation, sulfonation, and glucosylation. Moreover, *AtBAT1*-homologous genes, *At2g40230* and *At5g17540*, exhibit similar functions in plants [32]. Thus, in the present study, we obtained and analyzed transgenic creeping bentgrass plants overexpressing *AtBAT1*. As reported in Arabidopsis, we observed BR-deficient phenotypes in the transgenic plants, including semi-dwarf growth (Fig 2), shorter and wider leaves with a dark green color (Fig 3), and decreased number of vascular bundles (Fig 5). Considering the differences in leaf morphology between dicot and monocot plants, the shorter, wider, and greener leaves of the transgenic creeping bentgrass plants are similar to the small and round leaves of dark green color in Arabidopsis plants overexpressing *AtBAT1*. In addition, the short petioles observed in Arabidopsis plants might correspond to the reduced internode lengths in creeping bentgrass. Moreover, the lamina joint bending angle was smaller in the AtBAT1 plant than in the control plant (Fig 6), which is a well-known BR-deficient phenotype in the monocot. Therefore, the results of the present study demonstrate that expression of the *AtBAT1* gene can lead to similar BR-deficient phenotypes in both dicot and monocot plants (i.e., Arabidopsis and creeping bentgrass).

AtBAT1 is a member of the BAHD acyltransferase family, named after benzylalcohol O-acetyltransferase (BEAT), anthocyanin O-hydroxycinnamoyltransferase (AHCT), anthranilate N-hydroxycinnamoyl/benzoyltransferase (HCBT), and deacetylvindoline 4-O-acetyltransferase (DAT). The BAHD acyltransferases are known to utilize CoA thioesters and catalyze the formation of ester or amide bonds in plant metabolites [56]. The Arabidopsis genome contains 61 members of the BAHD acyltransferase family, which can be classified into five clades through phylogenetic analysis [57]. According to this classification, AtBAT1 is a member of clade V. In a previous phenotypic analysis of transgenic Arabidopsis plants overexpressing 13 genes in the clade, two genes (*At2g40230* and *At5g17540*) showed a similar function to *AtBAT1* [32]. In addition, another BAHD acyltransferase belonging to clade III (At4g15400), known as BIA1 (Brassinosteroid inactivator 1) or ABS1 (Abnormal shoot1-1), has shown to reduce the levels of BRs and cause dwarf phenotypes when overexpressed in plants [58, 59]. Therefore, it is apparent that members of the BAHD acyltransferase family play a role in maintaining BR homeostasis during plant growth and development by inactivating BRs via acyl conjugation. Indeed, overexpression of *AtBAT1* decreased the endogenous levels of BR intermediates of the late oxidation pathway, such as 6-deoxoTY, 6-deoxoCS, TY, and to a lesser extent, CS and BL [30, 31]. More importantly, *in vitro* enzymatic activity assays with recombinant AtBAT1 protein have demonstrated that BL, CS, and TY were acylated, but teasterone (TE) was not [31]. These results suggest that the BR intermediates with α-configuration of C-3 hydroxy group are supposed to be the substrates that can be acylated by AtBAT1, which include 6-deoxoTY, TY, 6-deoxoCS, CS, and BL (S3 Fig). Thus, TE was not acylated by AtBAT1 because of its β-configuration of C-3 hydroxy group. In the present study, we further analyzed the endogenous levels of BR intermediates in the transgenic creeping bentgrass plants with AtBAT1 using 4 available internal standards, and confirmed that the endogenous levels of 6-deoxoTY, 6-deoxoCS, and CS were decreased (Table 1). Therefore, our data suggest that AtBAT1 catalyzes its enzymatic reactions similarly in both Arabidopsis and creeping bentgrass, and that acylation of BRs might represent a general inactivation mechanism in dicot and monocot plants.

Since overexpression of AtBAT1 decreases the endogenous levels of BRs with C-3 in α-configuration in plants, the changes in the levels of other BR intermediates is also expected for a homeostatic regulation of BRs. Indeed, it has been shown that the endogenous levels of two BR intermediates, 6-deoxocathasterone (6-deoxoCT) and 6-deoxo-3-dehydroteasterone (6-deoxo3DT), were increased in AtBAT1-overexpressing Arabidopsis plants [30]. In addition, transcript analysis by microarray and quantitative PCR confirmed increases in the expression of BR biosynthetic genes, such as *ROT3* (At4g36380) and *BR6ox2* (At3g30180) [31]. ROT3 (ROTUNDIFOLIA 3) is involved in the conversion of TY to CS, and BR6ox2 (BRASSINOSTEROID-6-OXIDASE 2) is known to catalyze 6-deoxoCS to CS and CS to BL. Thus, it has been suggested that the transcriptional increases of these genes might result from the feedback regulation in response to the reduced levels of endogenous BRs. However, we could not perform the quantitative analysis of other BR intermediates and expression analysis of BR biosynthetic genes in creeping bentgrass. Thus, further studies will be necessary to elucidate the homeostatic regulation of BRs when AtBAT1 is overexpressed in creeping bentgrass.

Due to the function of BRs during plant growth and development, they can control several important agronomic traits such as plant architecture, seed yield, and stress tolerance [6, 7, 10, 13]. Therefore, reducing the levels of BRs would not be a good way of manipulating yields, because BR deficiency usually leads to dwarfism and reduced fertility [14, 34, 35]. However, slight decreases in BR levels could lead to crop improvements, owing to changes in plant architecture [6]. For example, a rice BR-deficient mutant (*osdwarf4-1*) shows limited defects in BR biosynthesis and plant morphology, and represents a new cultivar with an erect leaf phenotype and increased grain yield [12]. This is because the erect leaves permit greater penetration of light to lower leaves, thereby enhancing photosynthesis, and nitrogen storage for grain filling, which facilitates the dense planting of crops. In addition, semi-dwarf varieties have been suggested to enhance biomass production with improved harvest index [7]. In this regard, overexpression of BR-inactivating enzymes might represent a more useful approach to confer moderate effects of BR deficiency than knocking out BR-biosynthesis genes. Previously, we generated a dwarf creeping bentgrass through overexpression of an Arabidopsis β-glucosidase (AtBG1) that hydrolyzes glucose-conjugated abscisic acid (ABA) to produce active ABA [40]. Compared with the dwarf plant expressing *AtBG1*, the expression of *AtBAT1* led to a semi-dwarf phenotype (Fig 2). Moreover, the transgenic plants expressing *AtBAT1* showed erect leaf phenotypes (Fig 6). Together, the results of the present study suggest that overexpression of *AtBAT1* might be useful for the improvement of agronomic traits of crops.

In addition to BR-deficient phenotypes such as semi-dwarfism and erect leaves, the transgenic creeping bentgrass plants overexpressing *AtBAT1* showed delayed senescence and drought tolerance (Figs 7 and 8). Since delayed senescence has been observed in many BR-deficient and BR-insensitive mutants [4], the senescence phenotype of the AtBAT1 plant is consistent with the previous results. On the other hand, the drought-tolerant phenotype of the AtBAT1 plant is not an expected one, because the treatment with exogenous BRs has been known to increase tolerance to stresses in plants [2, 5, 9, 60]. However, a recent study showed that farnesylation of CYP85A2, a cytochrome P450 enzyme that catalyzes the last step in BR biosynthesis, is important for ER (endoplasmic reticulum) localization, which is necessary for its function [55]. Therefore, loss of either CYP85A2 or CYP85A2 farnesylation results in reduced BR accumulation, but strikingly, also improved drought tolerance via increased plant responsiveness to abscisic acid (ABA). Thus, it is suggested that reduced endogenous BRs might lead to hypersensitization of the plants to endogenous ABA, in turn, promoting their tolerance to stresses. This could be supported by our results that the AtBAT1 plants showed higher ratios of stomatal closing than the control plant (Fig 9). Thus, the present study supports the notion that the plants with reduced endogenous levels of BRs is hypersensitive to ABA, which can increase stomatal closing ratios resulting in drought tolerance.

Of note, semi-dwarf rice mutants with erect leaves exhibit prolonged tolerance to drought [61]. Thus, the drought tolerance observed in the AtBAT1 plants may also be attributed to the plant architecture, especially erect leaves that are shorter, wider, and thicker than the control plant (Figs 3 and 6). It is also possible that semi-dwarfism, with reduced growth rates and fewer vascular bundles, contributes to drought tolerance (Figs 2, 4 and 5). Thus, moderate manipulation of endogenous BRs, which can induce semi-dwarf plants with short, wide, and thick leaves, might improve tolerance to stresses. In this regard, the genetic manipulation of endogenous BRs using BR-inactivating enzymes may represent a novel method of developing crops with improved drought tolerance.

Collectively, the results of the present study demonstrated that overexpression of an Arabidopsis BR-inactivating enzyme reduced the endogenous levels of BRs in creeping bentgrass, which resulted in BR-deficient phenotypes, such as semi-dwarfism with erect leaves and delayed senescence. In addition, the transgenic plants showed tolerance to drought stress with the increased responsiveness to ABA. Thus, creeping bentgrass with *AtBAT1* developed in this study has beneficial traits for the cultivation of turfgrass. Therefore, the manipulation of BR levels using the *AtBAT1* gene might provide an effective means to improve agronomic traits of crops, including dicots and monocots.

## Supporting information

S1 Fig Genomic PCR analysis of transgenic creeping bentgrass plants overexpressing *AtBAT1*. (PDF)

S2 Fig Measurement of chlorophyll contents in creeping bentgrass plants. (PDF)

S3 Fig Illustration for the changes of BR intermediates by AtBAT1. (PDF)
