# Supplementary material for: Overexpression of Arabidopsis thaliana brassinosteroid-related acyltransferase 1 gene induces brassinosteroid-deficient phenotypes in creeping bentgrass
Source: PLoS One. 2017 Oct 30;12(10):e0187378. doi: 10.1371/journal.pone.0187378 (PMC5662239; doi:10.1371/journal.pone.0187378)
Supplement: S1 Fig — (PDF) [file pone.0187378.s001.pdf]

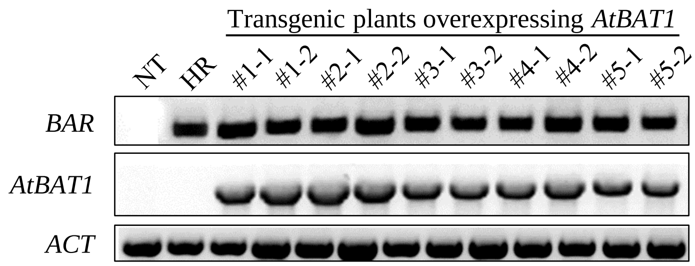

S1 Fig. Genomic PCR analysis of transgenic creeping bentgrass plants overexpressing *AtBAT1*. The coding regions of transgenes (*BAR* and *AtBAT1*) were PCR-amplified using genomic DNA from each plant as templates. The actin (*ACT*) gene of creeping bentgrass was also PCR-amplified and included as loading controls of the genomic DNA. NT, non-transformed control plant (i.e., a negative control); HR, transgenic plant with an empty vector (i.e., a positive control for *BAR*). After the transformation of creeping bentgrass, we randomly selected five independent transgenic lines, and genomic DNA was isolated from two plants of each line (10 transgenic plants in total), and also from NT and HR control plants. The first selected plants (#1-1, #2-1, #3-1, #4-1, and #5-1) were used as the representative plants of each transgenic line for further molecular analyses.
