# Supplementary material for: Overexpression of Arabidopsis thaliana brassinosteroid-related acyltransferase 1 gene induces brassinosteroid-deficient phenotypes in creeping bentgrass
Source: PLoS One. 2017 Oct 30;12(10):e0187378. doi: 10.1371/journal.pone.0187378 (PMC5662239; doi:10.1371/journal.pone.0187378)
Supplement: S2 Fig — (PDF) [file pone.0187378.s002.pdf]

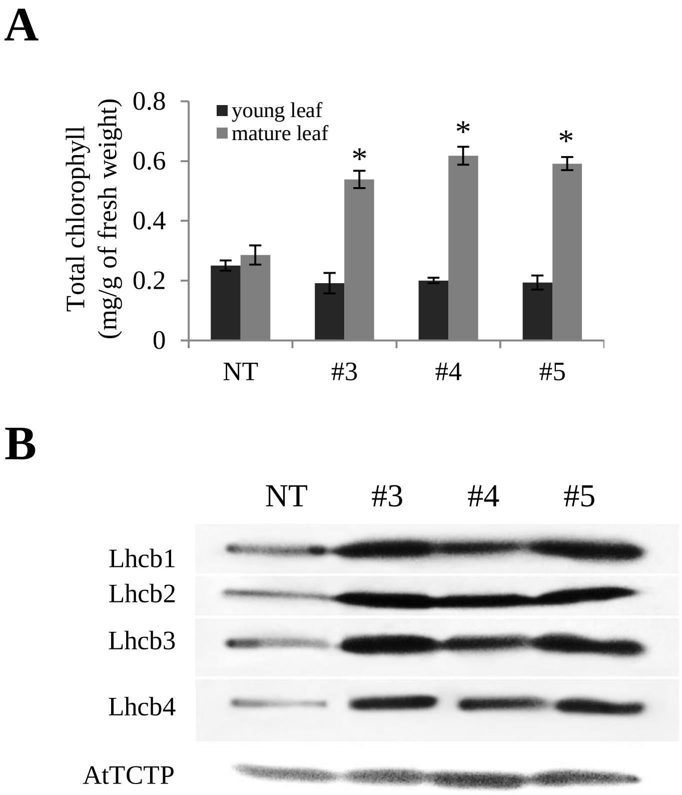

S2 Fig. Measurement of chlorophyll contents in creeping bentgrass plants. (A) Chlorophyll contents in young and mature leaves of non-transformed (NT) and transgenic AtBAT1 plants (#3, #4, and #5). Two-week-old leaves were harvested and used as young leaves, and leaves from 4-week-grown plants were used as mature leaves. Data represent means ± SD of three independent measurements. Statistically significant changes compared with NT are indicated by * at $P < 0.05$. (B) Western blot analysis of light-harvesting chlorophyll-binding (Lhcb) proteins. Mature leaves were used for this analysis. AtTCTP (*A. thaliana* translationally controlled tumor protein; At3g16640) was shown as loading controls.
