# Supplementary material for: Overexpression of Arabidopsis thaliana brassinosteroid-related acyltransferase 1 gene induces brassinosteroid-deficient phenotypes in creeping bentgrass
Source: PLoS One. 2017 Oct 30;12(10):e0187378. doi: 10.1371/journal.pone.0187378 (PMC5662239; doi:10.1371/journal.pone.0187378)
Supplement: S3 Fig — (PDF) [file pone.0187378.s003.pdf]

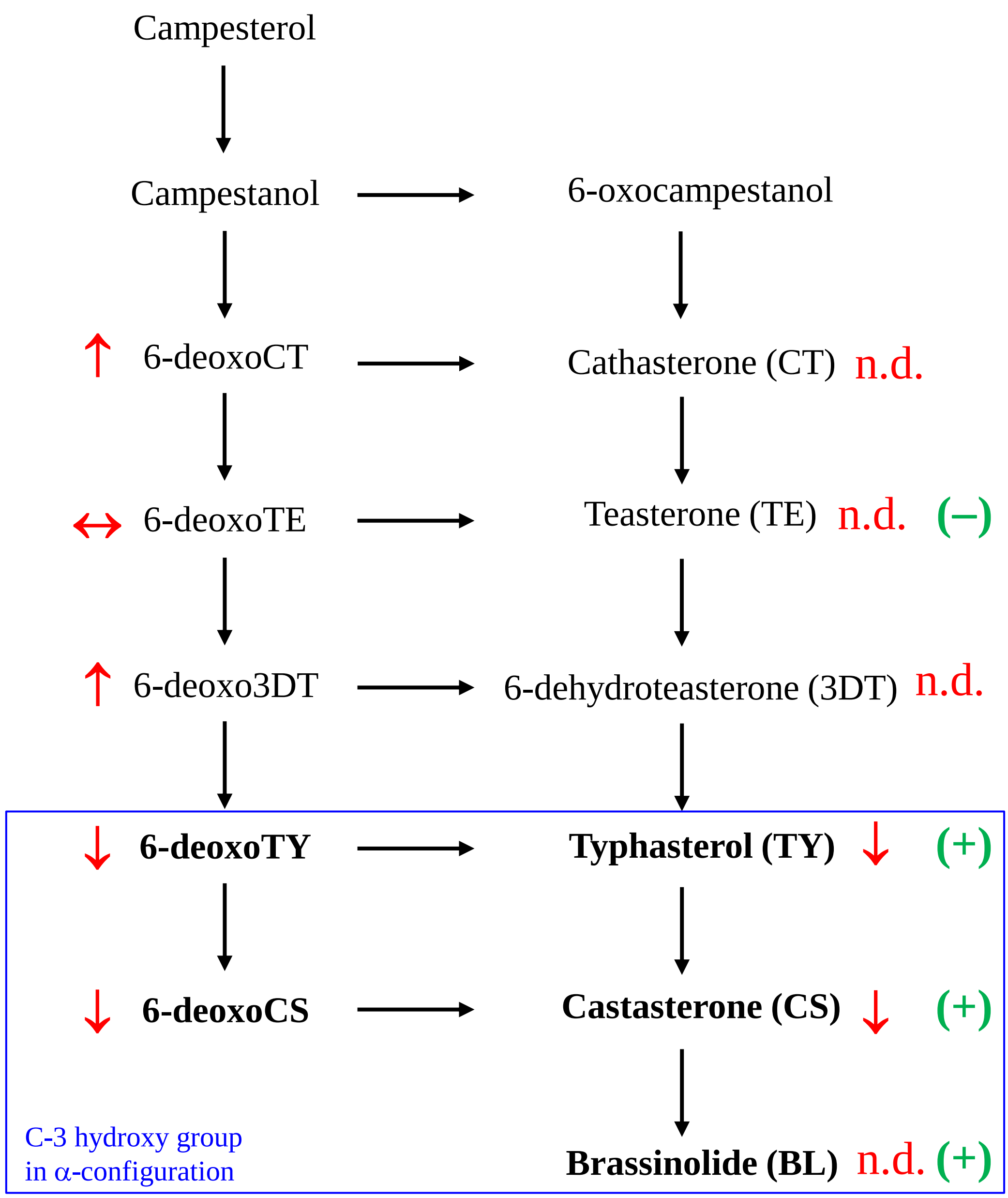

S3 Fig. Illustration for the changes of BR intermediates by AtBAT1. This summarizes the changes of BR intermediates in the biosynthetic pathway by the overexpression of AtBAT1 in Arabidopsis plants, based on previous reports (from references 30–32). According to the experimental results, BR intermediates with α-configuration of C-3 hydroxy group are supposed to be the substrates that can be acylated by AtBAT1 (five intermediates in blue box). Except BL that cannot be measurable in plants, the endogenous levels of 4 BR intermediates with C-3 in α-configuration (6-deoxoTY, TY, 6-deoxoCS, CS) were reduced in AtBAT1-overexpressing plants (shown as ↓). On the other hand, two intermediates (6-deoxoCT and 6-deoxo3DT) were increased (shown as ↑), and other intermediates were not detected (n.d.) or no change (shown as ↔). In addition, *in vitro* enzymatic activity assays showed that BL, CS, and TY with C-3 in α-configuration were acylated by recombinant AtBAT1 [shown as (+)], whereas TE with C-3 in β-configuration was not acylated [shown as (–)].
